# Improving routine use of clinical pathway decision support through integration of an EHR with a clinical library resource designed to provide evidence-based guidance within oncology workflows

**DOI:** 10.1186/s12913-024-11018-8

**Published:** 2024-05-01

**Authors:** Mary Ichiuji, Laura Asakura, Carol Cain, Nancy Aye, Tatjana Kolevska, David Chen, Farah Mohebpour Brasfield, Dinesh Kotak

**Affiliations:** 1 The Permanente Federation, 1 Kaiser Plaza, Oakland, CA 94612 USA; 2 Kaiser Foundation Health Plan & Hospitals, 1 Kaiser Plaza, Oakland, CA 94612 USA; 3 The Permanente Medical Group, 1950 Franklin St, Oakland, CA 94612 USA; 4 Southern California Permanente Medical Group, 393 East Walnut St, Pasadena, CA 91188 USA

**Keywords:** Oncology, Clinical pathways, Clinical decision support, Workflow, Implementation science

## Abstract

**Background:**

The rapid evolution, complexity, and specialization of oncology treatment makes it challenging for physicians to provide care based on the latest and best evidence. We hypothesized that physicians would use evidence-based trusted care pathways if they were easy to use and integrated into clinical workflow at the point of care.

**Methods:**

Within a large integrated care delivery system, we assembled clinical experts to define and update drug treatment pathways, encoded them as flowcharts in an online library integrated with the electronic medical record, communicated expectations that clinicians would use these pathways for every eligible patient, and combined data from multiple sources to understand usage over time.

**Results:**

We were able to achieve > 75% utilization of eligible protocols ordered through these pathways within two years, with > 90% of individual oncologists having consulted the pathway at least once, despite no requirements or external incentives associated with pathway usage. Feedback from users contributed to improvements and updates to the guidance.

**Conclusions:**

By making our clinical decision support easily accessible and actionable, we find that we have made considerable progress toward our goal of having physicians consult the latest evidence in their treatment decisions.

## Contributions to the literature

• Research has shown that consulting evidence-based treatment pathways for cancer can help physicians keep current with new scientific discoveries and leads to better patient outcomes.
• We designed treatment pathways that would be easily accessible to physicians by placing it in the electronic health record system, making it more efficient for them to order suggested treatments, and incorporated their input and the input of their peers so that they trusted the system.
• Through these design choices we were able to have > 90% of physicians use the pathways within 2 years, even though they were not required to.

## Background

Oncology treatment is complex and rapidly evolving, with a need for timely access to life-changing therapeutics matched to cancer stage and individual genomic considerations [1]. The use of care pathways in oncology has been shown to improve patient care outcomes [2–5]. Kaiser Permanente (KP) has a foundational mission to provide evidence-based, up-to-date, high quality, equitable care to our members. To support KP physicians in fulfilling this mission, KP created an organized approach toward oncology care pathways that incorporated organizational leadership, structured participation and feedback, novel integration of technology tools, and evaluation of effectiveness. We hypothesized that physicians would use these care pathways if they were trusted, easy to use, and integrated into clinical workflow at the point of care. Our goal was to make it preferable for physicians to access clinical decision support (CDS) for every patient, every time.

### Organizational background

Kaiser Permanente is an integrated healthcare delivery system caring for more than 12.6 M members with a presence in 8 states plus the District of Columbia. The system includes 39 hospitals, over 700 medical offices, an estimated 23,000 physicians and 320 oncologists [6]. The integrated nature of KP’s medical groups, hospitals, and health plan allows KP to design a proactive, coordinated model of care across clinical disciplines.

Cancer care has been an area of strategic clinical priority for KP due to the needs of cancer patients, the healthcare professionals caring for them, and the promising advancements in science. There is potential to dramatically affect clinical outcomes with appropriate early intervention, but keeping up with new information is challenging for individual physicians due to the rapid evolution of treatment innovations and the increasing need for specialization.

### Clinical goals and clinical leadership

KP is represented across 8 geographic regions by clinical specialty experts, in this case including specialists in medical, surgical, radiation oncology, and pharmacy. It became overwhelmingly apparent to leaders and practitioners that a single individual would find it challenging to keep pace with the knowledge, data, and analysis needed to deliver high quality care. At present, KP offers online reference resources through its Clinical Library as well as treatment protocols built in Epic Beacon, but had little active support for selection of protocols. Care pathways have been shown to be an effective method for communicating complex clinical guidance and improve patient outcomes, as the graphical decision-tree structure quickly synthesizes the guidance to reveal individual patient options [1]. Under the auspices of the Cancer Care strategy, multi-disciplinary oncology clinical leaders from across KP collaborated to produce KP Cancer Care pathways, setting up an internal process, governance structure, and tools. KP Cancer Care pathways are held to a high standard for considering the most current guidelines and evidence, emphasizing efficacy and safety, incorporating clinical stakeholder input, and updated regularly. The charter for the teams creating KP Cancer Care pathways explicitly states that finding, understanding, and using clinical guidance should be easier for the physician than their past practice of looking in reference materials and navigating order sets. After creating and implementing each pathway, the teams are charged to review user feedback, opening a line of communication with individual physicians. A total of 11 inter-regional, multi-specialty teams for different organ systems are currently organized to create care pathways and update them on a quarterly basis.

### Technology

KP uses the Epic Systems [7] Electronic Health Record (EHR) and since 2009 has used the Beacon Oncology module to house KP’s system-wide, standardized, evidenced-based oncology treatment protocols. Due to the size of the KP healthcare system and variations in local geographies, KP’s EHR is comprised of multiple instances of Epic Systems installations, with variations in discrete clinical data such as laboratory fields used. KP also independently supports a Clinical Library (KP-CL) system [8] – a curated internal electronic repository of clinical knowledge resources and tools, hosted on an Adobe Experience Manager [9] platform for content management and web services. KP-CL interfaces with the EHR through standard APIs provided by Epic Systems.

KP’s clinical, technology, and analytic experts were thus assembled to create, deploy and update KP Cancer Care pathways within clinical workflows.

## Methods

Kaiser Permanente, an integrated delivery system in the United States with approximately 320 oncologists, was interested in understanding whether an inter-regional program to author and deploy CDS for oncology treatment pathways would be utilized by oncologists for a majority of their patients. Use of the KP Cancer Care pathways was not required for initiating treatment for patients, so individual physicians are only incentivized by easier workflow and the ability to practice care based on the latest evidence. The pathway tools became available across the organization within a short time period, and we report usage and user feedback over a period of 3 years. The program components included the authoring of clinical guidance, creation and integration of a care pathway tool, and analysis of usage and user feedback.

### Clinical guidance

KP oncology leadership, after a thorough review of care pathway tools available for purchase externally, endorsed the creation of a KP-built CDS tool. A charter was established in January 2019 with the mission “to facilitate provision of the highest quality cancer care by developing expert level decision support at the point of care” [10]. Multi-disciplinary teams were convened to synthesize the best available scientific evidence and create care pathways for priority conditions. Where the evidence base exists, the teams have access to physicians and pharmacists who search the scientific literature and assist in formulating recommendations according to GRADE methodology  [11]. When the evidence base is not as robust, such as emerging therapeutics, the teams formulate consensus-based expert recommendations based on their own professional attendance at clinical meetings, discussion of available studies, and support from KP’s knowledge experts. In addition, KP’s evidence analysts provide context for the adoption of external guidance by evaluating the guideline development processes used by the most prominent groups in the field.

In each team, participants include medical oncologists, surgical oncologists, radiation oncologists, specialized pathologists, and pharmacists for the specialty as appropriate, drawn from a representative sample of KP’s geographic regions. Treatment pathways specific to cancer types were created with criteria regarding the stage of disease, genomic targets or immunotherapy score, bulky or limited disease, and patient comorbidities.

Pathway creation was prioritized based on the volume of patients or the clinical impact of providing high quality guidance. The co-chairs of KP’s inter-regional specialty groups prioritized the pathways for their specialty and, with the assistance of multi-disciplinary team members, created the first drafts of each pathway. These were then vetted by expert colleagues that represented their geographic area. Having local representation of respected experts added to the depth of knowledge and enhanced the trust that users had in the content. As with all KP-developed guidance, pathway authors considered the highest quality evidence, efficacy, and safety first, then patient preference and cost considerations, following the GRADE framework [11, 12] for developing clinical guidance. The teams aimed for guidance that was clear, simple, and direct, covering common clinical situations, with explanations if consensus-based guidance was utilized. This work built upon oncology treatment protocols that KP has programmed into Epic’s Beacon module by providing the larger context of disease progression and evaluation. Pathways created by the teams are formally vetted in each KP region. After consensus is reached amongst the specialty team, the pathways are reviewed by the governance committee (KP Inter-Regional Chiefs of Oncology) for approval. The system launched with 12 pathways in 2 specialties. The teams have created over 150 pathways to date, which are reviewed on a quarterly basis for clinical currency and to incorporate user feedback.

### Care pathway availability

**Fig. 1:**
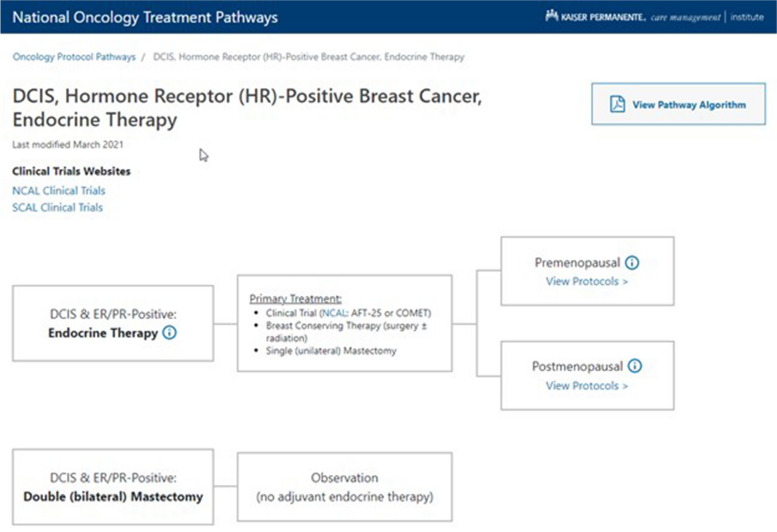
Sample care pathway

The oncology care pathways are published to KP-CL, a trusted, curated resource of clinical knowledge guidance and tools, accessed over 5000 times daily by KP clinicians. KP-CL includes both KP-authored content and subscriptions to external resources. While the majority of the resources are static documents in html and PDF format, the care pathways are published as graphical, clickable process diagrams, and includes clinical content available as hover text (Fig. 1). Meta-tags are associated with various components of the care pathway, including, most critically, the pathway component that the user clicks to return to the EHR. In addition, we capture standard data about page use, user characteristics and browsing context.

### Interface with epic systems EHR

KP-CL is available as a custom button within KP’s EHR interface, as well as through right-clicking and direct URL links. KP-CL uses a standard Active Guidelines interface to receive contextual information when accessed, including information about the user, patient, and encounter. Users typically access the oncology pathways when they are in the oncology module via the Navigator. Despite the multiple instances of the EHR system throughout KP, KP-CL is able to maintain one version of the oncology pathways by using a standard interface.

After a treating oncologist browses the KP-CL oncology pathway to arrive at a treatment recommendation they agree with, they click on that recommendation to order the protocol. With this click, KP-CL passes data back to EHR to order the specific treatment protocol via the standard protocol ordering workflow. As drug formularies and some operational protocol details may differ by region, each KP instance maintains a preference list that translates the general order from KP-CL into a region/state-specific protocol order. The oncologist experience is thus a one-click transition from pathway recommendation to EHR order, with regional differences handled in the background. The oncologist is then able to interact with and modify the order per standard EHR functionality.

### Deployment

This work builds upon clinical and technical foundations already present throughout KP, such as the regular meetings of inter-regional oncology leaders, their clinical communication channels with their peers, familiarity with KP-CL and EHR Oncology protocols, and an earlier, wiki-based trial in one KP region. KP-CL translated and created the graphical pathways corresponding to the clinical guidance, which was vetted by an oncology expert and tested by Epic application coordinators.

Because KP-CL is maintained centrally and uses standard Epic interfaces, technical deployment was relatively straightforward and occurred over a few weeks. By February 2020, all KP regions had access to the oncology pathway CDS. Technical go-live was accompanied by email communications from the region’s oncology chief or designated champion to all the region’s oncology users. Because the functionality is intuitive, no formal training was provided. No financial incentives were offered for using the tool.

The oncology chiefs agreed to adopt a Quality goal of achieving 70% usage of the pathways in patients whose cancer type is addressed in an available pathway. Oncology chiefs receive monthly reports about physician usage of pathways, with friendly competition amongst regions and medical centers to increase adoption. The chiefs also review user feedback.

### Analytics

To understand the impact of this CDS, we combined Epic EHR and KP-CL data. For each use of the pathway page on KP-CL, we collected information about the physician use and basic encounter context, as well as the pathway branch chosen and whether the preferred vs alternative option was chosen. The reports include pathways that have options for both early and advanced disease. In a few conditions, our pathways incompletely represent the disease spectrum, and because our analysis does not include the cancer stage of patients, we exclude the incomplete pathways from the report, since patients may be at a stage not captured by the pathway. We capture as the denominator the number of treatment protocols ordered in these common diagnoses, and the numerator as the number of protocols ordered through KP-CL CDS. We consider protocols “ordered” after the first instance of the drug is administered. For individual usage, we calculate a monthly denominator of unique providers entering oncology treatment plans, against the monthly numerator of unique providers who ordered at least 1 treatment plan via KP-CL.

### Feedback

Users of KP-CL pathways can give feedback that a pathway or protocol is not available for a specific patient’s circumstances. Users are also able to give unstructured feedback through a feedback button available on the CDS pathway home page. The information is sent via email message to the core CDS team. A core team member responds to this feedback within 48 hours, routing it to the appropriate accountable group. Clinically relevant requests are reviewed by the subspecialty teams to consider edits to the pathways. The inter-regional chiefs of oncology also receive monthly quantitative reports showing the percentage of protocols ordered via pathways as well as physician participation rates.

## Results

**Fig. 2:**
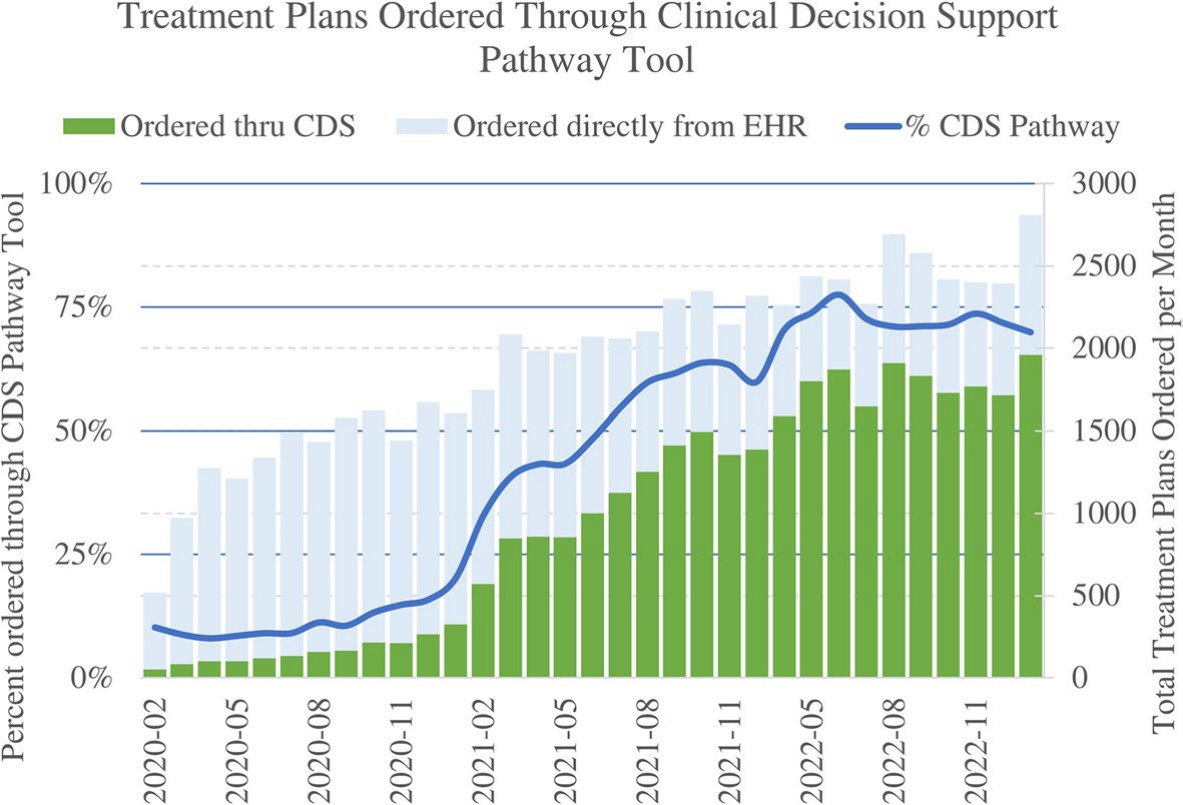
Oncology pathway CDS tool usage over time

KP oncologists order > 30,000 Beacon protocols annually in the clinical conditions supported by our pathways. We experienced dramatic organic uptake of oncology CDS, despite offering minimal training and no external incentives. From an initial start of about 9% in the first quarter of deployment in February 2020, we saw pathway-based ordering grow to over 70% of eligible treatment plans by April 2022, rising to and holding steady above 75% in April 2022 through January 2023. Figure 2 shows the increase in percentage of protocols ordered via the graphical pathway since inception.

We have developed pathways in high-volume conditions for all major organ systems where a majority of patients would follow the preferred or alternative recommendation. As of December 2022, we have 117 pathways in 38 cancer/hematology types; of these, we monitor utilization on the 99 pathways in 28 cancer types where we have complete treatment options for all stages of disease progression.

While our goal is for the pathways to be accessed for every patient, we do not have pathways for every diagnosis, nor do we believe that every patient must follow a particular pathway. As with all clinical guidance, we would not expect 100% of patients to follow the preferred recommendation, due to clinical variation and co-morbidity. While we have data related to users browsing KP-CL pathways, we are not able to validate that the browsing is related to selecting a treatment plan, and thus we do not attempt to capture the percent of users who consult the pathway without ordering from the pathway.

KP-created Epic Beacon protocols are groupings of cyclical treatments within a treatment plan. Each protocol contains 5–6 cycles. We thus estimate that ~ 100,000 treatments were ordered with the assistance of CDS. The most commonly used pathways in 2022 were for breast cancer, followed by lung cancer, colorectal cancer, and prostate cancer, accounting for 35% of the pathway volume consulted. This usage aligns with cancer prevalence in the population, which is what we would hope as we intend for pathways to be consulted for every patient, even if the pathway is not followed.

**Fig. 3:**
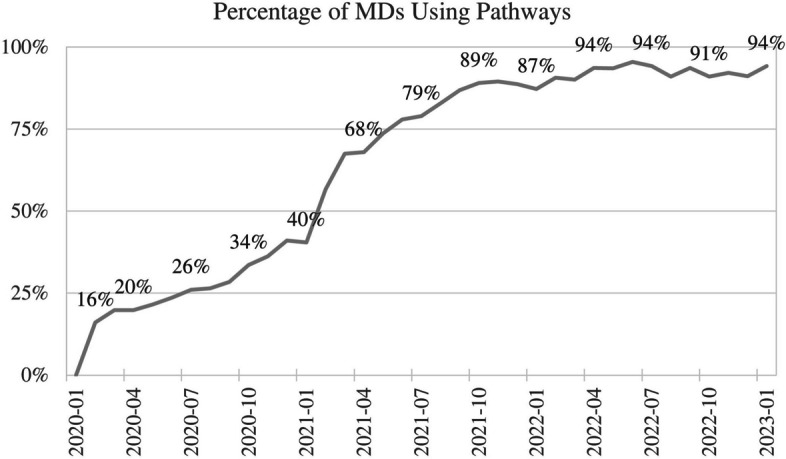
Percentage of individual oncologists ordering using CDS

We analyze the uptake of CDS over time by individuals to understand the effectiveness of our communication efforts (Fig. 3). While the CDS is available to all users immediately after launch, we monitor launch communications, specialty group meetings, and other trainings to assess whether further communication is necessary. After three years of deployment, we find that over 90% of KP’s approximately 320 oncologists have used a pathway to order a protocol.

KP physicians have given feedback on the pathways, reporting 76 comments from Feb 2020 through Dec 2022. The suggestions have resulted in improvements to the home page and pathway content. Seventeen of the comments were requests for 12 new pathways in different conditions, and 10 of them have subsequently been added. There were 19 requests for improvements to an existing pathway (such as through adding a protocol or separating clinical categories); these were forwarded to the appropriate subspecialty team for evaluation.

## Discussion

We find that we have made considerable progress toward our goal of having physician users consult clinical pathways decision support before ordering oncology protocols, achieving over 75% usage within two years’ time, with over 90% of oncologists ordering through pathways voluntarily. Having physicians access CDS before choosing treatment plans helps KP achieve its goal of delivering high-quality evidence-based care, as physicians are consulting with the aggregated expertise of KP’s specialists before making treatment decisions in a rapidly evolving field. The clinical pathways support, but do not replace clinical expertise, and while we expect that pathways would be followed a majority of the time, we would not expect 100% of patients to be on the preferred pathway.

In 2018, we had evaluated options for purchasing externally developed oncology care pathways. Because of KP’s integrated care model, existing physician expertise, and lack of prior authorization requirements, we decided to develop an internal pathways tool that would be intuitively simple for users and reflect KP’s standards for evidence-based care.

We believe that several factors contributed to rapid adoption of oncology pathway CDS at KP. First and foremost, this CDS system inspires trust from KP oncologists because we consult the expertise of KP oncology peers for the clinical content of the pathways. The clinical guidance encoded in the pathways is built by physician leaders representing every KP region, according to quality standards that mirror or exceed external guidelines [13]. The pathways incorporate feedback from multidisciplinary teams through a process that is transparent to all stakeholders. The experts constructing pathways are practicing physicians who use the pathways themselves, and who communicate regularly with their oncology peers. They are supported by a multi-disciplinary team of pharmacists, evidence analysts, informaticists, and other clinical specialists to interpret the data and literature.

Secondly, we designed this solution for intuitive use in routine care. Since KP physicians are not required to use pathways for preauthorization, we expected physicians would not use a cumbersome tool, and thus our design needed to be more efficient than what was available through the EHR. Formerly, oncologists needed to search for a protocol using key terms, then follow multiple steps to order it. We imposed design constraints on our pathways, including a maximum number of 3 additional clicks to order a protocol, graphical browsing of the clinical content, integration into clinical EHR workflow, and actionable decisions.

We also included visible and granular feedback mechanisms to enable continuous improvement and dialogue with all users. Users can free-text feedback to the clinical leadership team while interacting with the pathways, thus alerting us to gaps in treatment options or clinical updates. The specialty teams receive this feedback and review it quarterly to guide pathway updates, additions, and revisions. The teams also have access to regional reports of pathway utilization, which they use to understand not just CDS usage, but whether the preferred pathway is in fact chosen more frequently. The feedback results in clinical and operational improvement, while reinforcing user confidence that the KP pathway guidance reflects the best judgment of KP’s oncology experts.

By creating the pathways in KP-CL, we intend to reap several benefits. First, KP-CL is already a trusted source of clinical tools for KP physicians at the point of care. Housing the pathway in a single location means that we can update guidance centrally for all regions’ benefit, and thus becomes a powerful vehicle for change. Individual physicians tend to order protocols they are familiar with, since it is difficult to keep up with clinical developments. By using pathways CDS, which they know is maintained by KP’s clinical experts, they can supplement their own research and memory to keep current. We have also used pathways to communicate short-term alerts such as medication supply shortages, helping our organization to (literally) stay on the same page. This approach helps KP fulfill its commitment to equitable, up-to-date, high quality care.

### Limitations

Our analysis of pathway usage, derived from webpage visits, protocol ordered, and first drug administered, cannot provide a definitive picture of whether a patient ultimately received the complete therapy. We analyze a large, commonly used subset of our available pathways but are missing some pathways in our analysis because they are novel, rare, or do not address the complete disease spectrum. We do not link the patient’s diagnosis and cancer stage to the pathways used, so we cannot validate that the physician was using the appropriate disease pathway. While we have information that a physician visited the pathway for a particular patient, we have not yet done the analysis to verify that the treatment plan was completed, nor the clinical outcome for the patient.

Although we have set up the governance structure and maintenance cadence for this clinical guidance, keeping the clinical pathways up to date with scientific evidence remains a challenge. Since our subject matter experts are practicing physicians, they continue to maintain a clinical practice and leadership duties. Thus, while we do provide compensated administrative time to the pathway developers, they have only a few hours a month to devote to creating and updating pathways. We expect each physician expert to attend about 12 hours of group discussions annually, plus additional time for individual research and prework. To mitigate this burden, we supplement the physician expert team with pharmacists, pathologists, librarians, evidence analysts, and other content experts to review materials for physician decision-making.

### Future work

We have begun analysis of whether the user selected the preferred or alternative pathway. We plan to improve the analytics on pathway usage to tie it more directly to patient care, by analyzing data on the chosen pathway and mapping it to granular patient diagnoses to assess clinical appropriateness, prescribing concordance over time, and the clinical impact of increased pathway use through examination of treatment adherence and long-term outcomes.

We continue to expand the number of conditions covered by these treatment pathways, and have added 26 new pathways in 2023. Our current pathways focus on oncology drug treatment plans, but we will be expanding them to encompass comprehensive care pathways across the spectrum of cancer care from screening to diagnosis, treatment, and survivorship. Interfacing with Epic beyond Beacon treatment protocols will introduce technical complexity around ordering and clinical workflow. Our ambition is to include patients in care navigation, and thus our future roadmap includes actionable tracking systems with virtual navigation, decision support, patient reported outcomes, and innovations in virtual care. Our goal as we expand audiences for CDS remains the same: to ensure that users can quickly and easily access trusted, updated, evidence-based care.

## Conclusions

We find that we have made considerable progress toward our goal of having physician users consult clinical pathways decision support before ordering oncology drug treatment protocols. As with all clinical guidance, we would not expect physicians to follow the recommendation 100% of the time, due to clinical variation and co-morbidity, but we find that by making clinical pathways easily accessible and actionable within normal clinical workflow, physicians are consulting the latest evidence in their treatment decisions.

## Data Availability

The datasets used and/or analysed during the current study are available from the corresponding author on reasonable request.

## Abbreviations

CDS: Clinical Decision Support
EHR: Electronic Health Record
KP: Kaiser Permanente
KP-CL: Kaiser Permanente Clinical Library
